# Defining the pediatric response to SARS-CoV-2 variants

**DOI:** 10.3389/fimmu.2023.1200456

**Published:** 2023-05-25

**Authors:** Reanne M. Ho, Asha C. Bowen, Christopher C. Blyth, Allison Imrie, Tobias R. Kollmann, Stephen M. Stick, Anthony Kicic

**Affiliations:** ^1^ Wal-yan Respiratory Research Centre, Telethon Kids Institute, University of Western Australia, Nedlands, WA, Australia; ^2^ Medical School, University of Western Australia, Nedlands, WA, Australia; ^3^ Wesfarmers Centre of Vaccines and Infectious Diseases, Telethon Kids Institute, Nedlands, WA, Australia; ^4^ Department of Infectious Diseases, Perth Children’s Hospital, Nedlands, WA, Australia; ^5^ Menzies School of Health Research, Charles Darwin University, Darwin, NT, Australia; ^6^ School of Biomedical Sciences, University of Western Australia, Nedlands, WA, Australia; ^7^ Telethon Kids Institute, University of Western Australia, Nedlands, WA, Australia; ^8^ Department of Pediatrics, University of British Columbia, Vancouver, BC, Canada; ^9^ Department of Respiratory and Sleep Medicine, Perth Children’s Hospital, Perth, WA, Australia; ^10^ Centre for Cell Therapy and Regenerative Medicine, School of Medicine and Pharmacology, University of Western Australia and Harry Perkins Institute of Medical Research, Nedlands, WA, Australia; ^11^ School of Population Health, Curtin University, Perth, WA, Australia

**Keywords:** COVID-19, SARS-CoV-2, pediatric, children, emerging variants, molecular biology, cellular biology, age-specific response

## Abstract

The global population has been severely affected by the coronavirus disease 2019 (COVID-19) pandemic, however, with older age identified as a risk factor, children have been underprioritized. This article discusses the factors contributing to the less severe response observed in children following infection with severe acute respiratory syndrome coronavirus 2 (SARS-CoV-2), including, differing viral entry receptor expression and immune responses. It also discusses how emerging and future variants could present a higher risk to children, including those with underlying comorbidities, in developing severe disease. Furthermore, this perspective discusses the differential inflammatory markers between critical and non-critical cases, as well as discussing the types of variants that may be more pathogenic to children. Importantly, this article highlights where more research is urgently required, in order to protect the most vulnerable of our children.

## 1 Introduction

The current coronavirus disease 2019 (COVID-19) pandemic has become common knowledge to most, including the general community, and is caused by the severe acute respiratory syndrome coronavirus 2 (SARS-CoV-2). Studies have identified that ‘older age’ and being ‘male’ remain primary risk factors for infection (1, 2). These along with underlying comorbidities or respiratory illness have been suggested to possibly increase susceptibility to infection and severe disease (2, 3). Furthermore, children <14 years appeared to have significantly lower susceptibility to infection, with seroprevalence of SARS-CoV-2 increasing with age (4). However, a recent systematic review did note variability in the reporting of age-specific infection rates with most estimated attack rates (AR) in pediatric groups now being comparable to adults (5). If true, it raises the question as to why children appear less susceptible to SARS-CoV-2 infection and associated disease?

**Figure 1:**
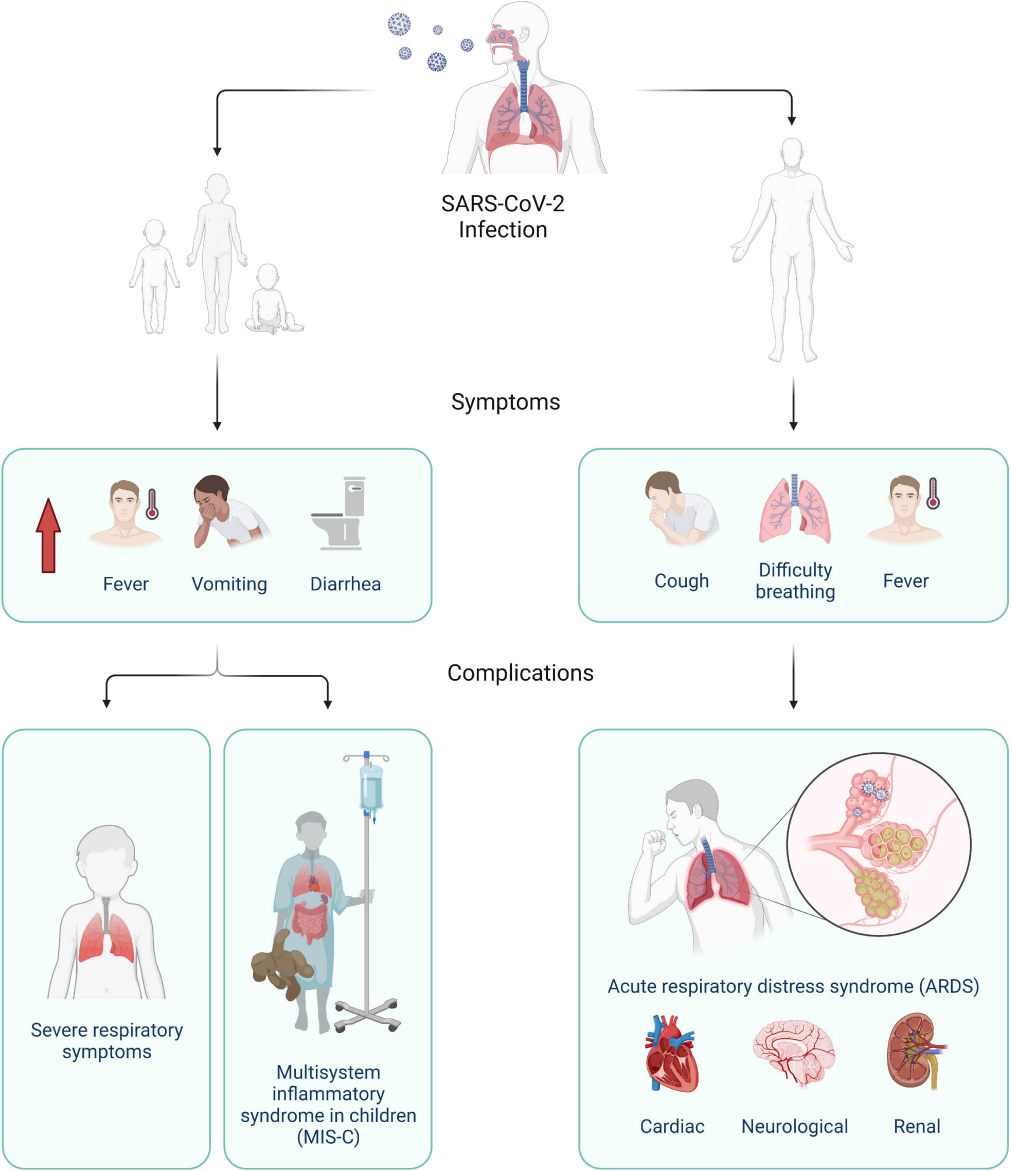
Clinical progression of SARS-CoV-2 infection in children (left) and adults (right). In children, higher proportion of fever, vomiting, and diarrhea were reported compared to adults and severe disease can manifest as severe respiratory symptoms or multisystem inflammatory syndrome (MIS-C), Adults typically present with common respiratory illness symptoms, which can lead to severe complications such as acute respiratory distress syndrome (ARDS) and as well as cardiac, neurological, and renal complications. Created with BioRender.com.

The currently known clinical spectrum of COVID-19 in adults is broad with about 80% of presentations being mild, 15% needing hospitalization, and 5% requiring intensive care (6). These rates still appear far lower in children (cumulative hospitalization incidence was 49.7 per 100,000 children) as the pandemic continues (7). The disease presents predominantly as a respiratory illness (**Figure 1**), but presentation can range from asymptomatic infection to extrapulmonary manifestations (3). A common subsequent complication includes acute respiratory distress syndrome (ARDS), but infection with SARS-CoV-2 may also lead to other types of organ failure and death (2, 3). Most children do not present with respiratory illness, but higher proportion of fever, vomiting, and diarrhea are observed upon admission to hospital (**Figure 1**) (2, 8). They are also at risk of a rare complication with non-specific symptoms and organ dysfunction termed COVID-19 associated multisystem inflammatory syndrome in children (MIS-C), also termed pediatric inflammatory multisystem syndrome temporally associated with SARS‐CoV‐2 (PIMS‐TS) (9–11). These differences suggest a possible deviation in the underlying immune response of adults and children to the virus.

## 2 Immune responses

Viral pathogens typically bind to host receptors, which then stimulate appropriate immune responses to control infection. However, since these receptors interact with the virus, they can also facilitate viral entry into cells. Consequently, one of the main factors thought to affect SARS-CoV-2 infection susceptibility is the expression of its receptors and relevant host factors (enzymes), namely angiotensin converting enzyme 2 (ACE2) and transmembrane protease serine 2 (TMPRSS2), in the airways (12, 13). Interindividual variations of distribution and expression of these receptors in the upper airway have been suggested to influence the infectivity of SARS-CoV-2 (12). This has been supported by several other studies where higher receptor expression is associated with increased age and the male sex (**Supplementary Table 1**).

Collectively, these observations suggest that the lack of respiratory symptoms in children might be due to a distinct infection course resulting from the difference in receptor expression. The lower expression of these host receptors in children could account for reduced viral entry and hence a less severe course of infection. However, it should be noted, that several studies have reported no correlation between age or sex with *ACE2* or *TMPRSS2* (14–16) (**Supplementary Table 1**). How “children” have been defined as part of study inclusion criteria may have also contributed towards these conflicting observations. Some have used the term to broadly encompass children and adolescents between 0-17 years of age (15–17), whereas others have utilized a restricted age range, i.e. <10 years (13, 18). This is a likely factor for the inconsistency seen for *ACE2* expression levels where younger children (<10 years) appear to have significantly lower *ACE2* expression expression compared to their older counterparts (10-17 years) (19). Thus, a more standardized approach in the terminology used for age cohorts or in the study design regarding the inclusion criteria will assist in interpreting results generated in future studies. Multiple other host receptors and factors with affinity to SARS-CoV-2 have also started to be implicated in the infection process by providing alternative entry pathways and enhancing viral entry (**Supplementary Table 1**). As this is an extensive list of potential receptors, there is still a lack of corroborated information on the relationship between their expression levels, especially in children and the potential effects they might have on susceptibility to SARS-CoV-2 infection.

**Figure 2:**
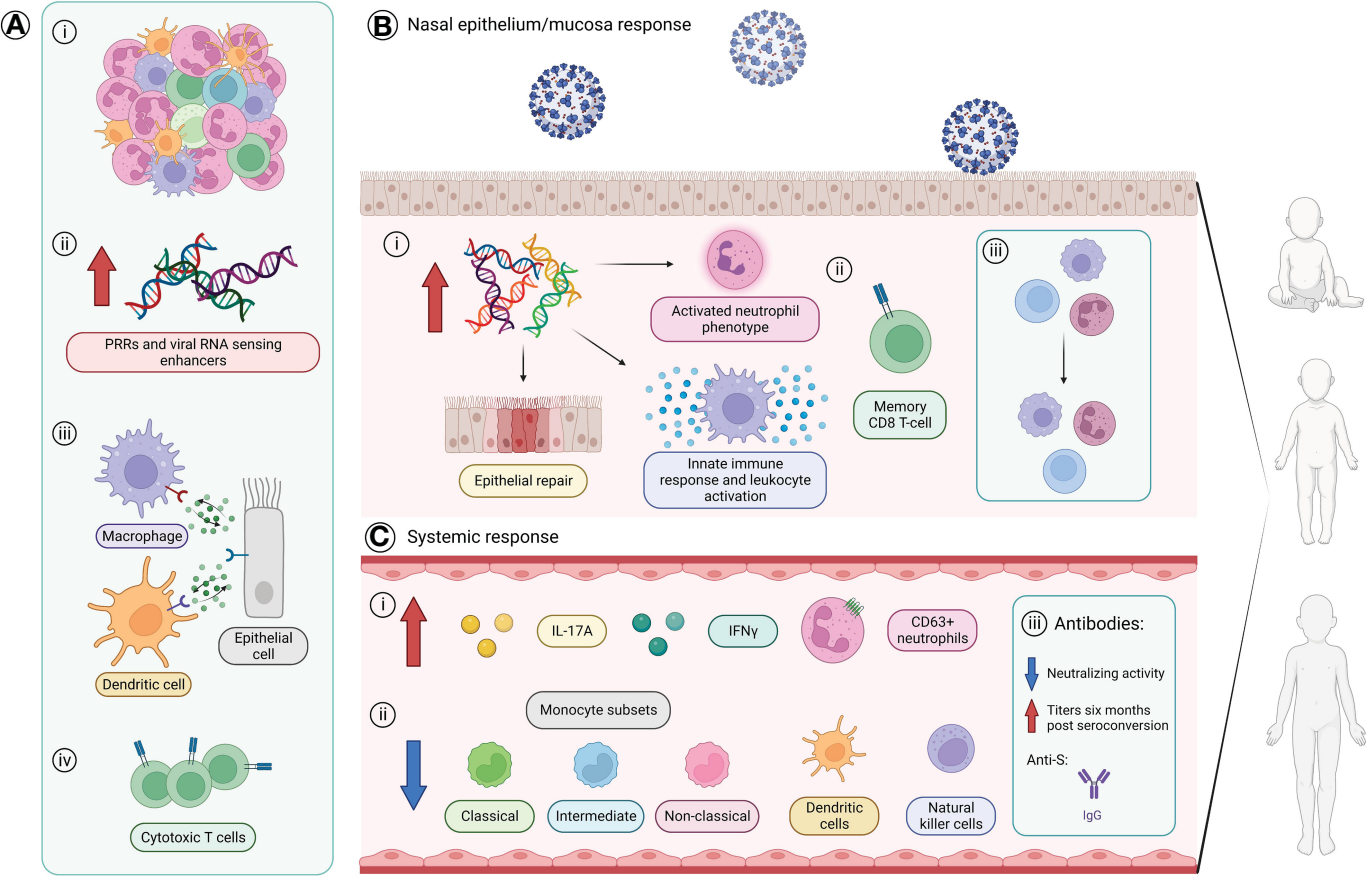
The age-specific innate and adaptive immune responses of children. **(A)** Baseline variances found in children compared to adults prior viral infection. **(i)** Greater number and diversity of immune cells with a neutrophil predominance. **(ii)** Higher levels of genes coding for SARS-CoV-2 pattern recognition receptors (PRRs) and viral RNA sensing enhancers. **(iii)** Stronger immune-epithelial cell cross-talk. **(iv)** Presence of a subpopulation of cytotoxic T-cells that allows preservation of virus-specific CD8^+^ T cell response without apoptosis. **(B)** Differences found in the nasal epithelium/mucosa immune response of children compared to adults following infection of SARS-CoV-2. Children are suggested to mount a stronger anti-viral response with: **(i)** increased gene expression levels involved with an activated neutrophil phenotype, epithelial repair, and innate immune responses such as response to TNF, leukocyte activation, and *IFNG* and *CCL5* expression, and **(ii)** presence of a distinct CD8^+^ T cell population with a memory phenotype. **(iii)** Stable maintenance of the proportion of immune and epithelial cells in nasal epithelium of children. **(C)** Systemic immune differences found in the blood of children compared to adults. **(i)** Higher levels of serum IL-17A and IFNγ and circulating CD63^+^ neutrophils. **(ii)** Reduced proportions of circulating monocytes subsets (classical, intermediate, non-classical), dendritic cells, and natural killer cells. **(iii)** Antibody response in children primarily consists of anti-S protein IgG antibodies with an overall lower neutralizing activity, but children maintain higher antibody titers against the S protein and RBD six months after seroconversion. Created with BioRender.com. IFN, interferon; IL, interleukin; N, nucleocapsid; RBD, receptor-binding domain; S, spike; TNF, tumor necrosis factor.

Studies have also investigated the initial innate immune responses of children compared to those of adults which have led to the discovery of multiple differences, both prior to and following SARS-CoV-2 infection (**Supplementary Table 2** and **Figure 2**). Amongst these, children appear to be capable of eliciting a more immediate and stronger anti-viral response when infected as shown by significantly higher levels of *IFNG* and *CCL5* expression in the nasal epithelium (15) (**Figure 2**). This stronger anti-viral response would allow for better control of SARS-CoV-2 replication in the airway and thereby, prevent the development of COVID-19-associated symptoms. This is in line with pediatric nasal epithelial cells being found to be less permissive to SARS-CoV-2 replication and mount a heightened antiviral response resulting in reduced viral replication following infection (20). Furthermore, lower viral loads were also found in upper respiratory tracts of asymptomatic children (<17 years) compared to children who present with symptoms (21). Interestingly, a very recent study by Koch and colleagues (16) did, however, report similar interferon gene responses in the nasal mucosa of children and adults infected with SARS-CoV-2.

SARS-CoV-2 infection in children also appears to reduce proportions of many immune cell types in the blood (**Supplementary Table 2**) (22–24). However, the observed higher serum IL-17 and IFNγ levels seen suggests that other cell types may be driving their production (22, 25). One such source may be CD8^+^IL-2^-^TNF^+^IFN-γ^+^ T-cells which have been found *via* flow cytometry to drive the SARS-CoV-2 T-cell response (26). Antibody responses also appear to differ between adults and children where, in children, the antibody response appears to be largely limited to anti-S IgG antibodies (**Figure 2**), unlike the broader response seen in adults (1). Furthermore, antibodies generated in children appear to have overall lower levels of neutralizing activity (1) (**Figure 2**). This appear in contrast to a recent study that reports similar antibody responses in seropositive children and adults to SARS-CoV-2 proteins (26). Distinct antibody profiles between children and adults are also seen with increased disease severity, where severely ill children with MIS-C show a broad non-specific IgG-driven monocyte-activating response, while adults with severe acute COVID-19 have enhanced IgA-related responses linked to neutrophil activation (27). As for the longevity of the humoral immunity, children have been reported to maintain higher antibody titers against the S protein and RBD compared to adults at least six months after seroconversion and these levels are only slightly reduced 12 months post-seroconversion (26). These results indicate the dire need for further studies to form an established understanding of the effects of age on the response to SARS-CoV-2 infection.

## 3 Multisystem inflammatory syndrome

In addition to severe COVID-19 typically seen in adults, SARS-CoV-2 infection may also drive a multisystem inflammatory response in children termed MIS-C or PIMS-TS (10, 22, 28, 29). Children who develop MIS-C are more likely to require intensive care with invasive ventilation, intravenous corticosteroids, vasoactive infusions, and inotropic support (11). They also typically present with leukocytosis with lymphopenia as well as high levels of systemic inflammation markers (24, 28). Work conducted has also identified lower lymphocyte counts in those with MIS-C compared to their non-MIS-C counterparts with acute presentations (25). Higher serum concentrations of IL‐1β, IL‐8, IL-10, TNFα, and IFNγ as well as more monocyte/antibody‐dependent cellular phagocytosis (ADCP) activity has also been associated with MIS-C (24, 25, 27, 29). Moreover, elevated pro-inflammatory cytokines (IL-6 and IL-17A) as well as chemokines (CXCL1, CXCL5, CXCL6, and CXCL11) including those involved with lymphocytic and myeloid chemotaxis and activation (CCL3 and CCL4, CCL19, and CXCL10) and mucosal chemotaxis (CCL20) were able to distinguish children with and without MIS-C (22). Variance in specific lymphocyte populations, namely lower CD56^lo^ NK cells and CD4^−^ T cells (mostly CD8^+^ T cells), have also been shown in MIS-C cohorts relative to infected non-MIS-C cohorts (10, 22).

Interestingly, similar antibody profiles have been exhibited in pediatric cohorts regardless of disease severity (with or without MIS-C) (1). This similarity may imply that the humoral immune response to the virus is not associated with MIS-C pathogenesis. However, Yonker et al. (9) did observe elevated IgM and IgG responses to the SARS-CoV-2 RBD and Bartsch et al. (27), observed higher SARS-CoV-2 S protein–specific IgM, IgG1 and IgA1 titers in children with severe MIS-C compared with mild MIS-C. Children with MIS-C were also found to have higher anti-S and anti-RBD IgG as well as anti-S IgA titers with higher neutralization activity compared with those that did not meet the criteria for MIS-C but had severe respiratory symptoms and required increased positive pressure support above their baseline (severe COVID-19) (30). Although differences in antibodies were not seen between the MIS-C and mild COVID-19 group (30) another study found higher titers of anti-S and anti-RBD IgG in children with MIS-C compared to children with COVID-19 (31). Variable levels of neutralizing antibodies were also found with the mild group (30). This suggests that children with MIS-C have greater capacity to more effectively neutralize SARS-CoV-2 compared to children with severe COVID-19 (30). Another distinction between the MIS-C and severe COVID-19 groups is higher TNFα and IL-10 proportions in the former (29). In addition, as autoreactive antibodies (with possible targets broadly found across tissues) have recently been observed with MIS-C, anomalous immune responses may also be promoted leading to systemic inflammation and multi-organ involvement (10, 22). These findings show that there are certainly specific mechanisms following SARS-CoV-2 infection that are involved with the divergence from the standard COVID-19 progression into the development of MIS-C, but there is still more to be investigated and their significance still needs to be validated.

## 4 Pediatric responses to SARS-CoV-2 variants

The combination of SARS-CoV-2 accumulating mutations in 1-2 nucleotides every month and the lack of effective containment strategies have given rise to the most recent issue of the pandemic, the emergence of new variants (**Supplementary Table 3**) (32). Work by Salleh, Derrick, and Deris (33) have performed an in-depth review of some of the earlier variants of concern (VoCs) which have all been suggested to have higher binding affinity to the human ACE2 receptor (except B.1.1.529 [Omicron]) and increased transmissibility (33, 34) (**Supplementary Table 3**); suggesting increasing adaptation of viral infectivity to humans but does not necessarily mean greater pathogenicity. Infection with B.1.617.2 (Delta) in primary Air Liquid Interface (ALI) cultures however, has revealed extensive nuclear damage and syncytial formation with increased replicative capacity compared to an ancestral Australian clinical strain suggesting possible increased pathogenicity (35). This is consistent with increased pathogenicity observed with Delta compared to a D614G-bearing isolate in a hamster model study (36). Aside from higher viral entry efficacies, B.1.1.7 (Alpha), B.1.351 (Beta), P.1 (Gamma), and Delta were also more resistant to the neutralizing activity of monoclonal antibodies and convalescent sera from recovered COVID-19 patients compared to the wildtype strain *in vitro* (33, 37, 38). Omicron has also been reported to escape clinically approved monoclonal antibodies, even in a combination which was highly potent against Delta (39). With multiple other variants identified since the original strain, the virus appears to be evolving, raising the potential for new variants to emerge in the future (32). Nonetheless, as the specific antigenicity of these variants have yet to be established, it also gives rise to the question of how each variant would differ in pathogenicity and respond to any vaccine or therapeutic approach.

Due to the sudden increase in incidence of SARS-CoV-2 infection in children with the emergence of new variants, there is still a current lack of evidence surrounding the response of the pediatric airway to them. Consequently, there is still some reliance on pre-prints for information specifically for the more recent variants, Delta and Omicron (40, 41). These studies compared variants and their respective effects on transmissibility and/or pathogenicity in children (**Supplementary Table 3**). The Delta variant, for instance, has been associated with higher incidence and increased hospitalization rates in children (7, 42, 43) (**Supplementary Table 3**). However, in a study conducted in UK comparing clinical outcomes in children infected during either an Alpha predominance or a Delta predominance, both variants were associated with similar median illness duration and symptoms experienced where most symptoms were short-lived and resolved within five days (44). Odds ratios (ORs) for some symptoms as well as the median symptom burden were only slightly higher with Delta compared to Alpha (44). This study suggested that these variants cause similar illness and symptoms and short duration. Consistent with this, another study reported that infection with Alpha or Delta in children ≤18 years was not associated with increased disease severity compared to non-VoCs (45). Findings implied that the higher hospitalization rates seen with the emergence of Delta were not a result of increased pathogenicity of the variant but simply due to the increased prevalence of infection. However, a case series in Tunisia reported that Delta causes critical outcomes in infants (<2 years) without any underlying conditions whose mothers were unvaccinated, where over half of the patients had pediatric ARDS and 20% ended with death (46). While this is not an overarching outcome of all pediatric SARS-CoV-2 infections, it emphasizes the potential risk new variants could bring.

Other studies on the Omicron variant have reported that vaccine effectiveness against Omicron infection is severely reduced compared to Delta and previous VoCs (39, 47). Interestingly, there was a difference in the preferred entry pathway whereby Omicron predominantly infects cells *via* endosomal fusion activated by cathepsin L (CTSL) and B (CTSB), whereas D614, Alpha, and Delta variants rely on cell surface fusion *via* TMPRSS2 proteolysis of the spike (39, 41, 47). Furthermore, Omicron does not heavily rely on the spike-mediated fusion *via* TMPRSS2, and so does not induce fusion with adjacent cells resulting in syncytia (39, 41, 47). Syncytia formation in pneumocytes has been associated with severe COVID-19, where this feature was found in majority of post-mortem samples from patients who died of COVID-19 (48). The lack of syncytia formation with Omicron’s etiology could possibly mean lower virulence compared to previous VoCs, which is in line with the milder outcomes and lower risk for severe illness observed in children following infection with Omicron (40, 49, 50) (**Supplementary Table 3**). Collectively, these results do imply that Omicron appears to be more transmissible compared to previous VoCs due to the overall higher expression of CTSL and CTSB in airway epithelial cells (12, 41).

With regards to possible cross-reactivity between variants, Dowell et al. (26) reports that children previously infected by the original strain of the virus, produced SARS-CoV-2 specific antibodies that were also cross-reactive to Alpha, Beta, and Gamma. Levels of these cross-reactive antibodies in children six months post-infection were also higher than that of adults (26). However, it should also be noted, that antibodies in all individuals showed reduced capacity to neutralize infection by live virus (26). Another study on a relatively small sample size reported higher lymphocyte and white blood cell counts but lower IL-6 levels in younger children <12 years old infected with Delta compared to those ≥12 years (51). Age cohorts also differed in antibody levels with lower anti-SARS-CoV-2 IgG and IgM found on admission, and lower IgG but higher IgM in in younger children at convalescence (51). Younger children were also associated with a higher asymptomatic rate and milder illness with lower incidence of severe cases, pneumonia, and respiratory failure reported (51). With new studies being performed, clinical impacts of these new variants on the pediatric population are becoming more evident, however, additional studies are still required to elucidate the potential cellular and molecular differences these variants would incite in children.

## 5 Impact of comorbidities

With certain comorbidities highlighted to increase the risk of susceptibility to the effects of SARS-CoV-2 infection in adults, it is important to know if children living with underlying health conditions are also at higher risk. Overall, children (and young adults <21 years) living with comorbidities have been found to be more likely to develop severe COVID-19 requiring critical care and COVID-19-associated death than those without, and this risk increases with the number of comorbidities they have (11, 52). Further insights can be made from a recent cross-sectional study conducted by Kompaniyets et al. (53), who examined data from 43,465 patients ≤18 years of age who presented to hospital with COVID-19. Results showed that 28.7% of children had an underlying medical condition, and this increased to 62.9% in those who were hospitalized. Observed comorbidities included asthma, neurodevelopmental disorders, anxiety and depressive disorders, and obesity (53). Although asthma was the most common risk factor observed, its adjusted risk ratio (aRR) for hospitalization was not amongst the highest at 1.23. Instead, type 1 diabetes was reported to have the highest aRR (4.6), followed by obesity (3.07), and congenital cardiovascular anomalies (2.12) (53). Children with type 1 diabetes and congenital cardiovascular anomalies were also reported to have the highest risk of developing severe COVID-19 (53). Other studies have also associated asthma, neurological, cardiovascular, and immunological/hematological disorders (immunocompromised), gastrointestinal disease, respiratory comorbidities, prematurity, obesity, and diabetes in children with higher risk of requiring hospital admission (11, 28, 52). This is also observed during the period where Delta was predominant, with underlying respiratory and endocrine disorders found more common in adolescents who were hospitalized with COVID-19 compared to age-matched controls (54). Age-stratification showed that chronic lung disease, neurologic disorders, cardiovascular disease, prematurity, and airway abnormality were risk factors for severe COVID-19 in infants and toddlers under 2 years (53, 55). For older aged children, diabetes mellitus and obesity were the predominant risk factors associated with severe COVID-19 (53, 55). As of writing this review, the underlying mechanisms as to why and how these specific comorbidities increase the risk of developing severe disease in children remain unknown, however, several theories are now emerging including the potential for the accumulation of viral mutations during a period of prolonged infectivity (21, 56).

## 6 Conclusions

In summary, we present a current overview on pediatric COVID-19 literature, particularly the differences suggested to contribute to the lower severity seen in children infected with SARS-CoV-2. Despite progress being made in identifying age-related immune response differences to infection, little is still known as to how and why, in some children, the disease progression leads to MIS-C and varies from what is typically seen with adults. With vaccines being made available for the younger population, it is encouraging that unexposed children will have vaccine-derived immunity, however many of our youngest remain vulnerable. Consequently, the priority now should include understanding how emerging variants will affect these individuals as well as identifying complementary therapeutics that can be adapted and tailored to the relevant variant at the time. With continued scientific advances, we will be able to fully comprehend the underlying biology of all SARS-CoV-2 variants and hopefully identify therapies for the youngest of our population where none currently exist, to keep our children safe.

## Author contributions

RH and AK conceived the review topic and initial structure. RH conducted literature review and drafted the manuscript. AK and AB provided extensive structural and editorial revisions and approved the final version for publication. CB, AI, TK and SS contributed to the structure and content, provided critical review, and approved the final version to be published. All authors contributed to the article and approved the submitted version.
