# Supplementary material for: Defining the pediatric response to SARS-CoV-2 variants
Source: Front Immunol. 2023 May 25;14:1200456. doi: 10.3389/fimmu.2023.1200456 (PMC10248061; doi:10.3389/fimmu.2023.1200456)
Supplement: Supplementary file 1 [file Table_1.docx]

Supplementary Material

Defining The Pediatric Response to SARS-CoV-2 Variants

# Supplementary Figures and Tables

**Supplementary Table 1**. List of host receptors and factors including proteases suggested to be involved with viral entry and observed association with age

| **Potential role** | **Host receptor/factor** | **Suggested function** | **Differential expression patterns in the airway epithelium of adults** | **Reference** |
| --- | --- | --- | --- | --- |
| Main receptor and protease | Angiotensin converting enzyme 2 (ACE2) | Engages with SARS-CoV-2 via the spike protein as a receptor for cellular entry | Higher expression of the *ACE2* gene was associated with increased age and the male sex | (1–4) |
|  |  |  | Reduced *ACE2* gene expression with age | (4–7) |
|  | Transmembrane protease serine 2 (TMPRSS2) | Cleaves the spike protein of SARS-CoV-2 to allow fusion of the viral and host cell membranes | Expression of *TMPRSS2* in the airways and lungs was also shown to increase with age | (2–4) |
|  |  |  | No significant differences in gene expression associated to age or sex | (4–6) |
| Postulated receptors | Basigin (BSG or CD147) | Secondary receptor to facilitate endocytosis | No significant differences in gene expression associated to age or sex | (5) |
|  | Glucose regulated protein 78 (GRP78 or heat shock protein A5 [HSPA5]) | May translocate to the plasma membrane where it can bind to the S protein and possibly initiate viral internalization | No significant differences in gene expression associated to age or sex | (5,8) |
|  | Neuropilin-1 (NRP1) | Bind to furin-cleaved S1 fragment of the spike protein | No significant differences in gene expression associated to age | (6,8) |
|  | Toll-like receptor 1 (TLR1) | Trafficking of TLR1/2 and TLR6/2 heterodimers from the cell membrane into the intracellular environment may be a gateway in which the virus can enter cells. Interactions between TLR1/6 and the S protein may play a role in the immunopathology resulting from unregulated TLR activation | Not available | (8) |
|  | TLR2 |  |  |  |
|  | TLR6 |  |  |  |
|  | TLR4 | Uncontrolled TLR4-mediated inflammation has been suggested to contribute to immunopathological consequences in COVID-19 patients |  |  |
|  | Dipeptidyl Peptidase-4 (DPP4 or CD26) | Co-receptor for ACE2 to mediate SARS-CoV-2 cellular entry | Not available | (8) |
| Proteases involved with S protein priming or ACE2 cleavage | Cathepsin L and B (CTSL and CTSB) | Cleave the S protein and facilitates viral entry via viral and endosomal membrane fusion | No significant differences in CTSL gene expression associated to age or sex | (5,9) |
|  | Furin | A proprotein convertase (PPC) that pre-activates and cleaves SARS-CoV-2 S protein | Not significantly associated with age | (7) |
|  | A Disintegrin and Metalloproteinase 17 (ADAM17) | Participate in ACE2 cleavage and shedding into the extracellular space | No significant differences in gene expression associated to age or sex | (5) |

**Supplementary Table 2.** Comparative immune responses to SARS-CoV-2 between children and adults

|  |  | **Lower** | **Higher** |
| --- | --- | --- | --- |
| **Prior to SARS-CoV-2 infection** |  |  | - Greater amounts and diversity of immune cells with neutrophils presenting as the predominant subset(10) - Higher basal expression levels of genes coding for SARS-CoV-2 PRRs and associated viral RNA sensing enhancers in epithelial cells of the upper respiratory tract(10) - Stronger immune–epithelial cell interactions with immune cells of higher activation status expressing higher levels of *TLR2*, *IFIH1*, *IL1B*, *IL8*, *TNF*, *CCL3*, and *CCL4*(10) - Increased expression levels of the interferon-gamma (IFNγ)- and CCL5-coding gene in *KLRC1* (NKG2A)^+^ cytotoxic T cells (CTL2) found predominantly in children, which has a role in sustaining virus-specific CD8^+^ T cell response while preventing excessive activation and apoptosis(10) |
| **Following infection** | Localized responses in airway epithelium | - The proportion of immune and epithelial cells remains stable in the upper airways of children (unlike the influx seen with adults), with neutrophils exhibiting an activated phenotype (enhanced expression of genes such as *CCL3* and *CXCR1/2*)(10) | - Increased expression of PRR genes in epithelial cells observed in children would be more apparent in early infection (0-4 days), but the expression would become comparable to that of adults by day 5(10) - Upregulation of genes involved with epithelial repair processes and distinct innate immune response-related processes such as response to TNF and regulation of leukocyte activation(11). |
|  | Systemic responses in the blood | - Lower serum IL-6 and TNα(12–14) - Reduced proportions of circulating monocytes subsets (classical [CD14^++^CD16^−^], intermediate [CD14^+^CD16^+^], non-classical [CD14^low^CD16^+^]), dendritic cells, and natural killer cells during the acute phase(15) - Lower and less robust T cell responses(13,16) - Decreased percentages of γδ T lymphocytes(12,17) - Lower Tcm-like SARS-CoV-2-specific CD8+ T cells(16) | - Higher serum concentrations of IL-17A and IFNγ(12–14) - Increase in the proportion of circulating CD63+ neutrophils involved in pro-inflammatory mediator release(15) - Possess a distinct CD8^+^ T cell population with a memory phenotype that was barely found in adults(10) - More Tscm SARS-CoV-2-specific CD8+ T cells(16) |

**Supplementary Table 3.** Emerging variants of interest and the pediatric responses

| **Variant** | **Notable differences and mutations from early reference strain** | **Pediatric response** |
| --- | --- | --- |
| D614G mutation | Alteration of an aspartic acid to a glycine at amino acid position 614 (D614G) located at the surface of the S protein(18) | - Not included |
| B.1.1.7 (Alpha) | Possesses a deletion at positions 69 and 70 of the S protein (Δ69-70) and eight other mutations in the S protein including N501Y (within the RBD) and D614G(19,20) | - Estimated reproduction numbers (R) in children and teens increased from well below 1 with the initial variants to just slightly below and around 1 for children and teens, respectively(21) - Transmission rates and incidence in Israeli children (0-9 years) doubled compared to that of former strains, though hospitalization rates decreased slightly(22) |
| B.1.351 (Beta) | Has multiple nonsynonymous mutations, including D614G and some within the RBD (K417N, E484K, and N501Y)(18) | - Not available |
| P.1 (Gamma) | 12 missense mutations in the S protein including D614G and some within the RBD (K417N, E484K, and N501Y)(23,24) | - Children infected with Gamma were reported to have higher risks for hospitalization (OR: 5.9), requiring respiratory support (8.3), and severe disease as classified by the WHO Clinical Progression Scale (7.7)(25) |
| B.1.617.2 (Delta) | Share some mutations with B.1.351 and B.1.1.7 but also have new mutations in the S protein such as L452R, T478K, and P681R(18,23,24) | - Increased incidence and prevalence in children under 17 years(26,27) - Higher proportion of infected children under 19 years of age compared to those infected with the Alpha variant(27,28) - Increased hospitalization rates in USA, with a spiked increase in weekly hospitalization rates in children and adolescents in August 2021(29) - Higher viral loads in ALI cultures observed after infection with Delta compared to an ancestral strain(30); however viral loads observed in pediatric ALI cultures were significantly lower than that of adults |
| B.1.1.529 (Omicron) | Characterized by 45 amino acid substitutions, some shared with other VoCs, compared to one of the earliest sequences of the virus, 30 of which are located within the spike protein (15 in the RBD)(31)  Consists of subvariants including BA.1, BA.2, BA.3, BA.4, and BA.5(32) | - Milder outcomes following infection with Omicron were implicated with an Omicron cohort (children and adults) found overall less at risk than the Delta cohort for requiring visits to the emergency department or hospitalization within three days of infection identification(33) (pre-print) - Omicron cases in children reported to have highest admission frequency compared to Alpha and Delta, but were associated with lower rates of severe illness including MIS-C(34) - Proportion of infants in hospitalization increased during the Omicron-predominant era while proportion of teenagers decreased(34) - Children and adolescents <18 years of age comprised 27.7% (18.9% are <12 years) of patients infected during the period of Omicron emergence compared to 31.2% (22.3%, <12 years) during the predominance of Delta(33) (pre-print) - In children <5 years, monthly incidence rate of infection increased with the emergence of Omicron but risks for severe clinical outcomes were lower with Omicron compared to Delta(35) - Lower viral loads following infection with an ancestral strain and Delta observed in pediatric nasal epithelial cells compared to adult cells are not replicated with Omicron(30) |

**References:**

1. Wark PAB, Pathinayake PS, Kaiko G, Nichol K, Ali A, Chen L, et al. ACE2 expression is elevated in airway epithelial cells from older and male healthy individuals but reduced in asthma. Respirology. 2021 May 17;26(5):442–51. doi:10.1111/resp.14003

2. Muus C, Luecken MD, Eraslan G, Sikkema L, Waghray A, Heimberg G, et al. Single-cell meta-analysis of SARS-CoV-2 entry genes across tissues and demographics. Nat Med. 2021 Mar 1;27(3):546–59. doi:10.1038/s41591-020-01227-z

3. Saheb Sharif-Askari N, Saheb Sharif-Askari F, Alabed M, Temsah MH, Al Heialy S, Hamid Q, et al. Airways Expression of SARS-CoV-2 Receptor, ACE2, and TMPRSS2 Is Lower in Children Than Adults and Increases with Smoking and COPD. Mol Ther - Methods Clin Dev. 2020 Sep 11;18:1–6. doi:10.1016/j.omtm.2020.05.013

4. Sungnak W, Huang N, Bécavin C, Berg M, Queen R, Litvinukova M, et al. SARS-CoV-2 entry factors are highly expressed in nasal epithelial cells together with innate immune genes. Nat Med. 2020 May 1;26(5):681–7. doi:10.1038/s41591-020-0868-6

5. Aguiar JA, Tremblay BJM, Mansfield MJ, Woody O, Lobb B, Banerjee A, et al. Gene expression and in situ protein profiling of candidate SARS-CoV-2 receptors in human airway epithelial cells and lung tissue. Eur Respir J. 2020 Sep 1;56(3):2001123. doi:10.1183/13993003.01123-2020

6. Plaas M, Seppa K, Gaur N, Kasenõmm P, Plaas M. Age- and airway disease related gene expression patterns of key SARS-CoV-2 entry factors in human nasal epithelia. Virology. 2021 Sep 1;561:65. doi:10.1016/J.VIROL.2021.05.012

7. Scagnolari C, Bitossi C, Viscido A, Frasca F, Oliveto G, Scordio M, et al. ACE2 expression is related to the interferon response in airway epithelial cells but is that functional for SARS-CoV-2 entry? Cytokine. 2021 Apr 1;140:155430. doi:10.1016/J.CYTO.2021.155430

8. Gadanec LK, McSweeney KR, Qaradakhi T, Ali B, Zulli A, Apostolopoulos V. Can SARS-CoV-2 virus use multiple receptors to enter host cells? Int J Mol Sci. 2021 Feb 1;22(3):1–35. doi:10.3390/ijms22030992

9. Padmanabhan P, Desikan R, Dixit NM. Targeting TMPRSS2 and Cathepsin B/L together may be synergistic against SARS-CoV-2 infection. PLOS Comput Biol. 2020 Dec 8;16(12):e1008461. doi:10.1371/JOURNAL.PCBI.1008461

10. Loske J, Röhmel J, Lukassen S, Stricker S, Magalhães VG, Liebig J, et al. Pre-activated antiviral innate immunity in the upper airways controls early SARS-CoV-2 infection in children. Nat Biotechnol. 2022 Mar 18;40(3):319–24. doi:10.1038/s41587-021-01037-9

11. Koch CM, Prigge AD, Anekalla KR, Shukla A, Do Umehara HC, Setar L, et al. Age-related Differences in the Nasal Mucosal Immune Response to SARS-CoV-2. Am J Respir Cell Mol Biol. 2022 Feb 3;66(2):206–22. doi:10.1165/rcmb.2021-0292OC

12. Gruber CN, Patel RS, Trachtman R, Lepow L, Amanat F, Krammer F, et al. Mapping Systemic Inflammation and Antibody Responses in Multisystem Inflammatory Syndrome in Children (MIS-C). Cell. 2020 Nov 12;183(4):982-995.e14. doi:10.1016/j.cell.2020.09.034

13. Pierce CA, Preston-Hurlburt P, Dai Y, Aschner CB, Cheshenko N, Galen B, et al. Immune responses to SARS-CoV-2 infection in hospitalized pediatric and adult patients. Sci Transl Med. 2020 Oct 7;12(564):eabd5487. doi:10.1126/scitranslmed.abd5487

14. Consiglio CR, Cotugno N, Sardh F, Pou C, Amodio D, Rodriguez L, et al. The Immunology of Multisystem Inflammatory Syndrome in Children with COVID-19. Cell. 2020 Nov 12;183(4):968-981.e7. doi:10.1016/j.cell.2020.09.016

15. Neeland MR, Bannister S, Clifford V, Dohle K, Mulholland K, Sutton P, et al. Innate cell profiles during the acute and convalescent phase of SARS-CoV-2 infection in children. Nat Commun. 2021 Feb 17;12(1):1084. doi:10.1038/s41467-021-21414-x

16. Rowntree LC, Nguyen THO, Kedzierski L, Neeland MR, Petersen J, Crawford JC, et al. SARS-CoV-2-specific T cell memory with common TCRαβ motifs is established in unvaccinated children who seroconvert after infection. Immunity. 2022 Jul 8;55(7):1299-1315.e4. doi:10.1016/j.immuni.2022.06.003

17. Carter MJ, Fish M, Jennings A, Doores KJ, Wellman P, Seow J, et al. Peripheral immunophenotypes in children with multisystem inflammatory syndrome associated with SARS-CoV-2 infection. Nat Med 2020 2611. 2020 Aug 18;26(11):1701–7. doi:10.1038/s41591-020-1054-6

18. Salleh MZ, Derrick JP, Deris ZZ. Structural Evaluation of the Spike Glycoprotein Variants on SARS-CoV-2 Transmission and Immune Evasion. Int J Mol Sci 2021, Vol 22, Page 7425. 2021 Jul 10;22(14):7425. doi:10.3390/IJMS22147425

19. Volz E, Mishra S, Chand M, Barrett JC, Johnson R, Geidelberg L, et al. Assessing transmissibility of SARS-CoV-2 lineage B.1.1.7 in England. Nature. 2021 May 13;593(7858):266–9. doi:10.1038/s41586-021-03470-x

20. Meng B, Kemp SA, Papa G, Datir R, Ferreira IATM, Marelli S, et al. Recurrent emergence of SARS-CoV-2 spike deletion H69/V70 and its role in the Alpha variant B.1.1.7. Cell Rep. 2021 Jun 29;35(13):109292. doi:10.1016/J.CELREP.2021.109292

21. Ratmann O, Bhatt S, Flaxman S. Implications of a highly transmissible variant of SARS-CoV-2 for children. Arch Dis Child. 2021 Sep 1;106(9):e37–e37. doi:10.1136/ARCHDISCHILD-2021-321903

22. Somekh I, Stein M, Karakis I, Simões EAF, Somekh E. Characteristics of SARS-CoV-2 Infections in Israeli Children During the Circulation of Different SARS-CoV-2 Variants. JAMA Netw Open. 2021 Sep 1;4(9):e2124343–e2124343. doi:10.1001/JAMANETWORKOPEN.2021.24343

23. Faria NR, Mellan TA, Whittaker C, Claro IM, Candido DDS, Mishra S, et al. Genomics and epidemiology of the P.1 SARS-CoV-2 lineage in Manaus, Brazil. Science. 2021 May 21;372(6544):815. doi:10.1126/SCIENCE.ABH2644

24. Hirotsu Y, Omata M. Discovery of a SARS-CoV-2 variant from the P.1 lineage harboring K417T/E484K/N501Y mutations in Kofu, Japan. J Infect. 2021 Jun 1;82(6):276–316. doi:10.1016/j.jinf.2021.03.013

25. Edward PR, Lorenzo-Redondo R, Reyna ME, Simons LM, Hultquist JF, Patel AB, et al. Severity of Illness Caused by Severe Acute Respiratory Syndrome Coronavirus 2 Variants of Concern in Children: A Single-Center Retrospective Cohort Study. J Pediatric Infect Dis Soc. 2022 Oct 25;11(10):440–7. doi:10.1093/jpids/piac068

26. Siegel DA, Reses HE, Cool AJ, Shapiro CN, Hsu J, Boehmer TK, et al. Trends in COVID-19 Cases, Emergency Department Visits, and Hospital Admissions Among Children and Adolescents Aged 0–17 Years — United States, August 2020–August 2021. MMWR Morb Mortal Wkly Rep. 2021;70(36):1249–54. doi:10.15585/MMWR.MM7036E1

27. Elliott P, Haw D, Wang H, Eales O, Walters CE, Ainslie KEC, et al. Exponential growth, high prevalence of SARS-CoV-2, and vaccine effectiveness associated with the Delta variant. Science. 2021 Dec 17;374(6574). doi:10.1126/science.abl9551

28. Twohig KA, Nyberg T, Zaidi A, Thelwall S, Sinnathamby MA, Aliabadi S, et al. Hospital admission and emergency care attendance risk for SARS-CoV-2 delta (B.1.617.2) compared with alpha (B.1.1.7) variants of concern: a cohort study. Lancet Infect Dis. 2022 Jan 27;22(1):35–42. doi:10.1016/S1473-3099(21)00475-8

29. Delahoy MJ, Ujamaa D, Whitaker M, O’Halloran A, Anglin O, Burns E, et al. Hospitalizations Associated with COVID-19 Among Children and Adolescents — COVID-NET, 14 States, March 1, 2020–August 14, 2021. Morb Mortal Wkly Rep. 2021;70(36):1255. doi:10.15585/MMWR.MM7036E2

30. Zhu Y, Chew KY, Wu M, Karawita AC, McCallum G, Steele LE, et al. Ancestral SARS-CoV-2, but not Omicron, replicates less efficiently in primary pediatric nasal epithelial cells. Sugden B, editor. PLOS Biol. 2022 Aug 1;20(8):e3001728. doi:10.1371/journal.pbio.3001728

31. Willett BJ, Grove J, MacLean OA, Wilkie C, De Lorenzo G, Furnon W, et al. SARS-CoV-2 Omicron is an immune escape variant with an altered cell entry pathway. Nat Microbiol. 2022 Jul 7;7(8):1161–79. doi:10.1038/s41564-022-01143-7

32. Hadfield J, Megill C, Bell SM, Huddleston J, Potter B, Callender C, et al. Nextstrain: real-time tracking of pathogen evolution. Kelso J, editor. Bioinformatics. 2018 Dec 1;34(23):4121–3. doi:10.1093/bioinformatics/bty407

33. Wang L, Berger NA, Kaelber DC, Davis PB, Volkow ND, Xu R. Comparison of outcomes from COVID infection in pediatric and adult patients before and after the emergence of Omicron. medRxiv. 2022 Jan 2;2021.12.30.21268495. doi:10.1101/2021.12.30.21268495

34. Bahl A, Mielke N, Johnson S, Desai A, Qu L. Severe COVID-19 outcomes in pediatrics: An observational cohort analysis comparing Alpha, Delta, and Omicron variants. Lancet Reg Heal - Am. 2023 Feb 1;18:100405. doi:10.1016/j.lana.2022.100405

35. Wang L, Berger NA, Kaelber DC, Davis PB, Volkow ND, Xu R. Incidence Rates and Clinical Outcomes of SARS-CoV-2 Infection With the Omicron and Delta Variants in Children Younger Than 5 Years in the US. JAMA Pediatr. 2022 Aug 1;176(8):811–3. doi:10.1001/JAMAPEDIATRICS.2022.0945

**
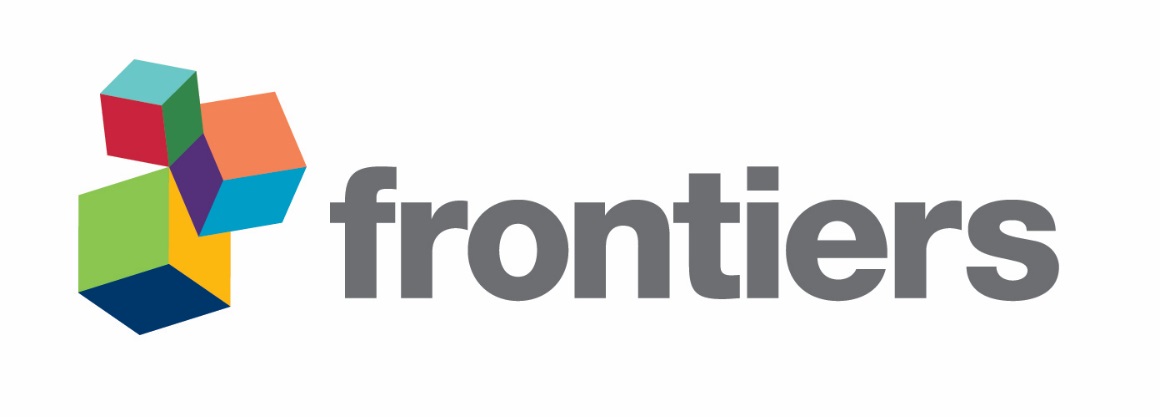
**
